# Dynamic Behavior of Reciprocating Plunger Pump Discharge Valve Based on Fluid Structure Interaction and Experimental Analysis

**DOI:** 10.1371/journal.pone.0140396

**Published:** 2015-10-21

**Authors:** Guorong Wang, Lin Zhong, Xia He, Zhongqing Lei, Gang Hu, Rong Li, Yunhai Wang

**Affiliations:** 1 School of Mechatronic Engineering, Southwest Petroleum University, Chengdu, Sichuan, PR China; 2 SJ Petroleum Machinery Co., China Petrochemical Corporation, Jingzhou, Hubei, PR China; UC Santa Barbara, UNITED STATES

## Abstract

The influence of spring stiffness and valve quality on the motion behaviors of reciprocating plunger pump discharge valves was investigated by fluid structure interaction (FSI) simulation and experimental analysis. The mathematical model of the discharge valve motion of a 2000-fracturing pump was developed and the discrete differential equations were solved according to FSI and results obtained by ANDINA software. Results indicate that spring stiffness influences the maximum lift, the opening resistance and shut-off lag angle, as well as the fluid velocity of the clearance, the impact stress and the volume efficiency of the pump valve in relation to the valve quality. An optimal spring stiffness parameter of 14.6 N/mm was obtained, and the volumetric efficiency of the pumping valve increased by 4‰ in comparison to results obtained with the original spring stiffness of 10.09N/mm. The experimental results indicated that the mathematical model and FSI method could provide an effective approach for the subsequent improvement of valve reliability, volumetric efficiency and lifespan.

## Introduction

Reciprocating pumps have been widely applied in the petrochemical, gas and general industry fields. Reciprocating pump operation is closely related to the efficiency and reliability of the suction and discharge valves. The valve is a key component of a reciprocating pump, which directly influences the performance and lifespan of the reciprocating pump. It has been proved that poor valve motion is the primary cause of valve failure and high flow resistance [1, 2]. Two factors greatly influence the valve motion and the subsequent fatigue and impact stress which acts upon the valve: valve quality and spring stiffness. The valve quality refers to the quality of the valve body testing by electronic balance. Therefore, a reasonably quality of pump valve and spring stiffness does not only extend the service life of a 2000-fracturing plunger pump, but also improves pump volumetric efficiency. Thus, the parameters optimization of valve quality and spring stiffness has a significant impact on the kinematics analysis of a reciprocating pump valve.

Many previous works have been conducted to investigate the valve dynamics of reciprocating pumps. Some studies have given greater focus to numerical simulations of valve motions. In 1968, U. Adolph determined the second order nonlinear differential equation of valve movement; however, this equation is only suitable to describe valve motion after the opening of the valve, rather than addressing the opening and closing processes of the valve [3–5]. The effects of the structure of the reciprocating compressor valve on established life and movement laws have been previously addressed by Xiao, who optimized valve structure from the spring and valve disc cone angle [6]. Movement of the valve when exposed to unsteady flow was studied by L. Boswirth, whose results indicate that a theoretical model alone cannot accurately describe the actual working condition because of fluctuations in gas pressure, mutation of the internal structural shape and changes in flow [7, 8]. Simulation analyses of valve motion indicate that springs and the Westphal phenomenon both influence the volumetric efficiency of the reciprocating pump. Moreover, a great spring stiffness can easily cause impact damage to the valve body and seat [9, 10]. Dynamic modeling of the flow process inside pressure regulating and shut-off valves was investigated by B.K. Saha, et al. using a computational fluid dynamic approach [11]. This method was able to predict spool movement and the final spool position when the spool position deviates from equilibrium; higher friction coefficients between the valve body and the spool were determined to be associated with quicker spool stability. Wang, et al. conducted an experimental investigation of valve impact velocity and the inclining motion of reciprocating compressors [12]. Results indicate that the inclining motion is negligible in the process of valve opening, while severe inclining motion was observed while the discharge valve was closing. When the pressure ratio exceeds 2.55, the inclining angle becomes uncertain due to the short time span encompassed by the discharge process. Furthermore, this experimental study provides a useful method for both valve testing and the optimization of valve reliability. Choi, et al. previously discussed the dynamic behavior of the valve system in a linear compressor based on fluid-structure interaction [13]. Numerical analysis indicated that the discharge valve opened when the piston compressed approximately 85% of the full stroke volume under the conditions of conical spring pre-load of 32N. As the pre-load was increased, the timing of the discharge valve opening was observed to be independent of the pre-load, though the discharge valve closure accelerated. An optimal pre-load of 48N was determined for the conical compression spring in order to achieve pump efficiency and reasonable impact stress. Wang and Liu have conducted some studies regarding the influence of spring stiffness on the performance of pump valves [14, 15], which indicate that spring stiffness has a significant influence on the motion characteristics of a reciprocating pump. A proper spring with optimized stiffness should be selected in order to achieve a balance between the pressure loss and leakage; previous literature indicates that a large spring stiffness results in increasing valve losses, whereas a small spring stiffness cannot efficiently seal the cylinder from the discharge chamber [16]. However, there is very little research which investigates the movement law of reciprocating plunger pump discharge valves from the perspective of optimized design of spring stiffness through FSI and experimental verification.

In this study, FSI and experimental analysis are conducted to study the motion law of discharge valves in order to improve the volume efficiency and stability of the reciprocating plunger pump through the optimized design of the spring stiffness and valve quality parameters.

## Theoretical Analysis of Valve Motion

**Fig 1:**
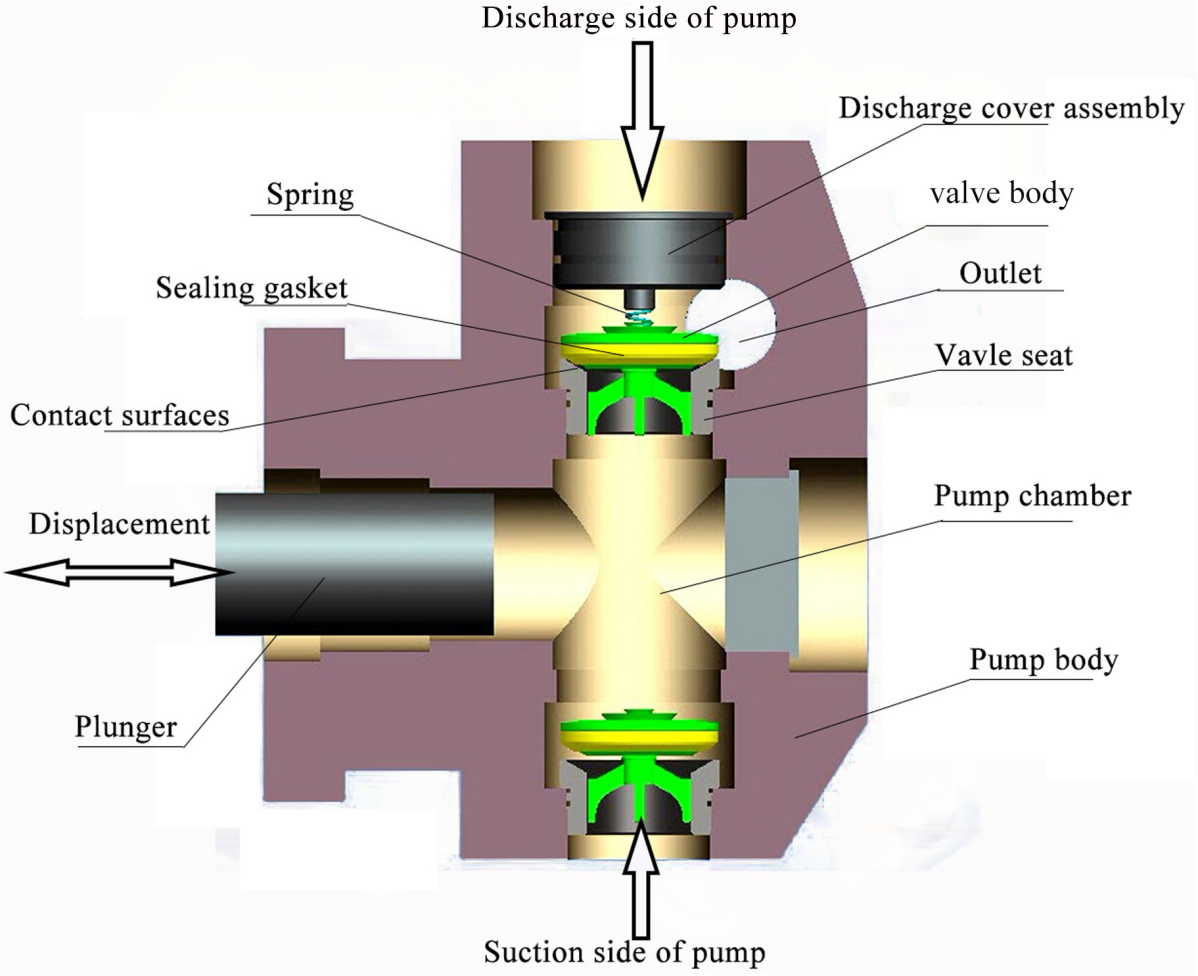
Schematic diagram of fluid end of a reciprocating pump.

The working principle of a plunger pump fluid end depends on a changing medium volume of sealed pump chambers achieved by the reciprocating movement of the plunger, thus realizing the suction and discharge operation of the pump valve. The valve itself consists primarily of spring, valve body, rubber sealing gasket and valve seat, as shown in Fig 1. This analysis primarily investigates the discharge valve motion of the plunger pump.

### 2.1 Mathematical model of valve motion

**Fig 2:**
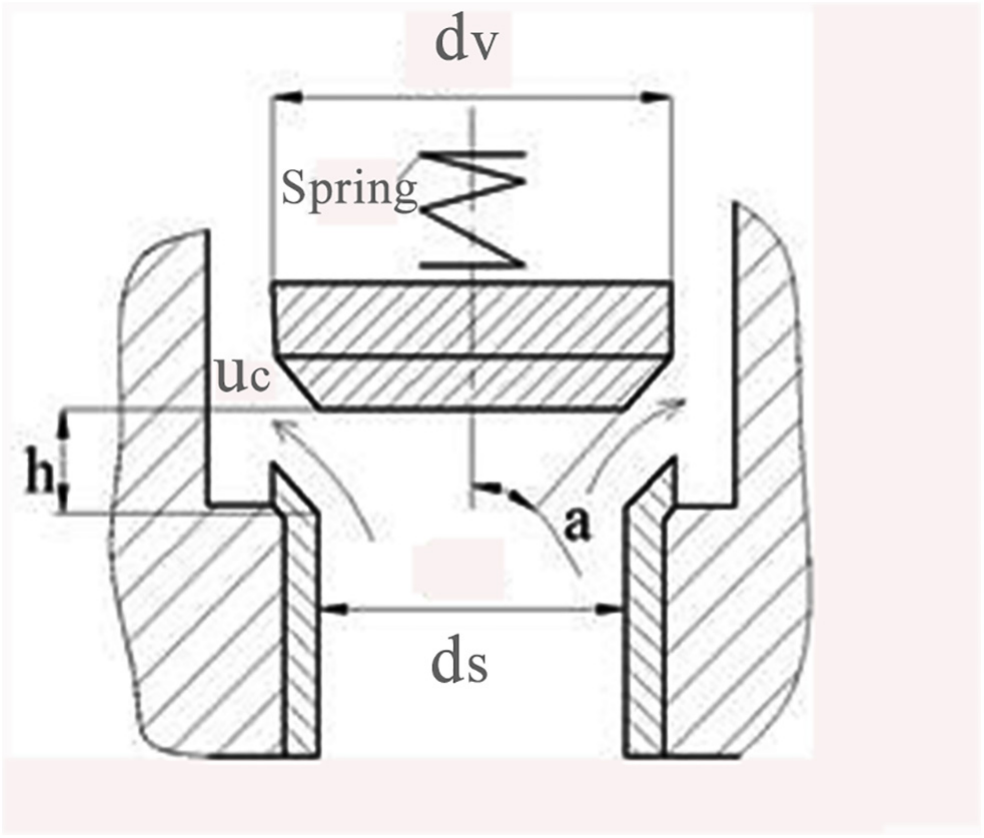
Schematic diagram of discharge side of a pump valve.

The structural diagram of the reciprocating pump discharge valve is depicted in Fig 2. According to fluid mechanics and the structure of the valve [17, 18], the continuous flow of valve clearance is given as follows:

$$Q_{c}=a\prime A_{c}u_{c}$$ (1)

where *Q*_*c*_ is the instantaneous flow of valve clearance; *a*′ is the contraction coefficient of the cross-sectional surface; and *u*_*c*_ is the fluid velocity of valve clearance. A value of *a*′≈1 was assumed in the present work, and A_c_ represents the sectional area of valve clearance. All the symbolic meanings of theory models are listed in Table 1.

**Table 1:** Nomenclature.

Nomenclature		h	Valve lift
Ǫ	Instantaneous flow	α	Angle between the axis and mating surface of valve
A_P_	Area of plunger end face	m	Valve quality
A_c_	Sectional area of valve clearance	ξ	Local resistance factor
*u*_p_	Sliding velocity of plunger	c	Spring stiffness
Ρ	Fluid medium density	F_0_	Pre-load of spring
*u*_c_	Liquid velocity of valve clearance	G	Valve body weight
P_1_	Fluid pressure below valve	a _v_	Valve body acceleration
P_2_	Fluid pressure above valve	Fs	Spring force
V_1_	Liquid velocity below valve	∆P	Pressure difference
V_2_	Liquid velocity above valve	Ǫ_C_	Instantaneous flow of valve clearance
Kr	Resistance coefficient	d_v_	Valve diameter
*a*′	Contraction coefficient of area	ds	Valve seat diameter
g	Acceleration of gravity	A_v_	Valve equivalent area
*u*_*v*_	Valve velocity	O _v_	Position of the valve body
Z_2_	Liquid surface height below valve	Z_1_	Liquid surface height above valve
V _b_	Velocity of valve body	ds	Valve seat diameter
X _v_	Displacement of valve body	X_m_	Displacement of plunger end face
V _f_	Fluid velocity of valve clearance	P _e_	Outlet pressure of pump fluid end
F _d_	Upper fluid force of discharge valve	F _u_	Lower fluid force of discharge valve

The Bernoulli equation of the actual fluid is expressed as follows:

$$\frac{P_{1}}{\rho g}+\frac{V_{1}^{2}}{2g}+Z_{1}=\frac{P_{2}}{\rho g}+\frac{V_{2}^{2}}{2g}+Z_{2}+K_{r}$$ (2)

where P_1_ is liquid surface pressure above the valve body; P_2_ is the liquid surface pressure below the valve body; ρ represents the density of the liquid medium; g is the acceleration of gravity; V_1_ is the liquid velocity below the valve body; V_2_ is the liquid velocity above the valve body; z_1_ is the liquid surface height below the valve body; z_2_ is the liquid surface height above valve body; and Kr is the coefficient of resistance.

The fluid resistance coefficient can be expressed as follows:

$$K_{r}=\frac{\Delta P}{\rho g}=\xi \frac{V_{c}^{2}}{2g}$$ (3)

where ∆P is the difference in pressure; and ξ is the local resistance factor. Considering the Westphalia phenomenon [1–4], the medium flow of valve clearance is expressed as follows:

$$Q_{c}=A_{p}u_{p}-A_{v}u_{v}$$ (4)

where Q_c_ is the instantaneous flow of valve clearance. The value of μ_v_ is positive when the valve rises, and negative when the valve drops.

**Fig 3:**
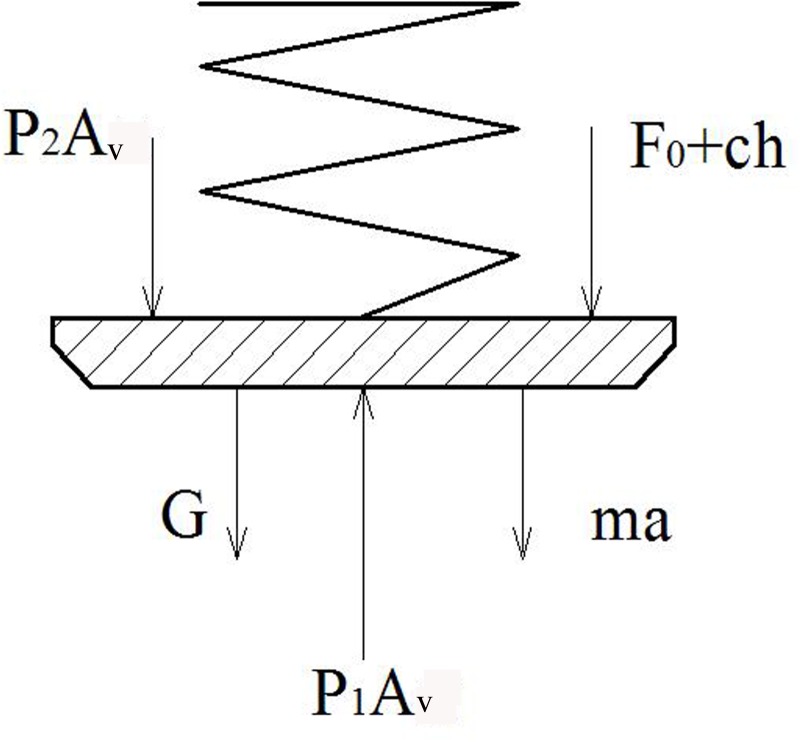
Schematic diagram of forces applied to valve.

Fig 3 depicts a schematic diagram of the forces acting on the valve, in consideration of the effects of inertia. The balance equation of the valve is expresses as follows:

$$A_{v}P_{1}=A_{v}P_{2}+G+F_{0}+Ch_{\left(t\right)}+\text{ma}$$ (5)

The mathematical model of valve movement can be deduced from Eqs (1)–(5) as follows:

$$\frac{m}{c}\ddot{h\left(t\right)}h{\left(t\right)}^{2}+h{\left(t\right)}^{3}+\frac{G+F_{0}}{c}h{\left(t\right)}^{2}+\frac{\xi \rho A_{v}^{2}A_{p}u_{p}}{c{\left(\pi d_{v}sin\alpha \right)}^{2}}\dot{h\left(t\right)-}\frac{\xi \rho A_{v}^{3}}{2c{\left(\pi d_{v}sin\alpha \right)}^{2}}{\dot{h\left(t\right)}}^{2}-\frac{\xi \rho A_{v}A_{p}^{2}u_{p}^{2}}{2c{\left(\pi d_{v}sin\alpha \right)}^{2}}=0$$ (6)

where m represents the valve quality; c is the spring stiffness; G represents gravity; F_0_ is the pre-load of the spring; α is the angle between the axis and mating surface of the valve; d_v_ is the diameter of the valve (see Fig 2).

Under discharge pressure conditions, the volumetric efficiency of plunger pumps is a volume ratio between the practical excretion liquid volume and the pump stroke volume in a discharge stroke.

The volume efficiency of plunger pumps under discharge pressure conditions is as follow:

$$\eta_{v}^{\prime }\;=1-\frac{\nabla V_{a}+\nabla V_{d}}{V_{h}}-\frac{\nabla V_{g}}{V_{h}}-\frac{\nabla V_{I}}{V_{h}}$$ (7)

Where, ∇*V*_*a*_ is the quantity of reflux induced by a suction valve closure lag, ∇*V*_*d*_ is the quantity of reflux caused by a discharge valve shut-off lag, *V*_*h*_ is the pump stroke volume, ∇*V*_*g*_ is the volume loss of liquid compression, ∇*V*_*I*_ is the volume loss of the liquid seal leakage.

Under the actual working conditions of the plunger pumper, the compressed of the liquid is relatively smaller and inevitable, and ignoring the seal leakage between the plunger and the cylinder. So the quantity of reflux, which is caused by the closure lag of the suction and discharge valve, is the main factors of the volume loss. Moreover, reducing the closing lag angle of suction and discharge valve can effectively improve the volumetric efficiency of pumps. Finally, the optimization design of the volumetric efficiency becomes the optimization of the lag angle.

Only the effect of the discharge valve closure lag angle on volumetric efficiency is considered, the volume efficiency under discharge pressure conditions is simplified as follow:

$$\eta_{v}^{\prime }=cos\varphi_{0}$$ (8)

where *φ*_0_ is the discharge valve closure lag angle.

### 2.2 FSI simulation of valve motion

When the hydraulic end of the fracturing pump operates under normal working conditions, a significant nonlinear response of valve motion may occur due to the combined effects of the high-speed impact of the flow field and the spring force, resulting in damage to the pump valve. FSI analysis of valve motion will determine the appropriate structure for optimized spring stiffness and valve quality. A model of the hydraulic end of the pump valve was developed by inputting valve motion as a nonlinear fluid-structure interaction system. Next, the structural and fluid models were established using computation solid and fluid dynamics. Meanwhile, the grid changes of the fluid-structure interaction interface were documented by the Arbitrary Lagrange-Euler (ALE) method, which is able to successfully simulate actual experimental conditions.

#### 2.2.1 Fluid-structure interaction model of valve motion

**Fig 4:**
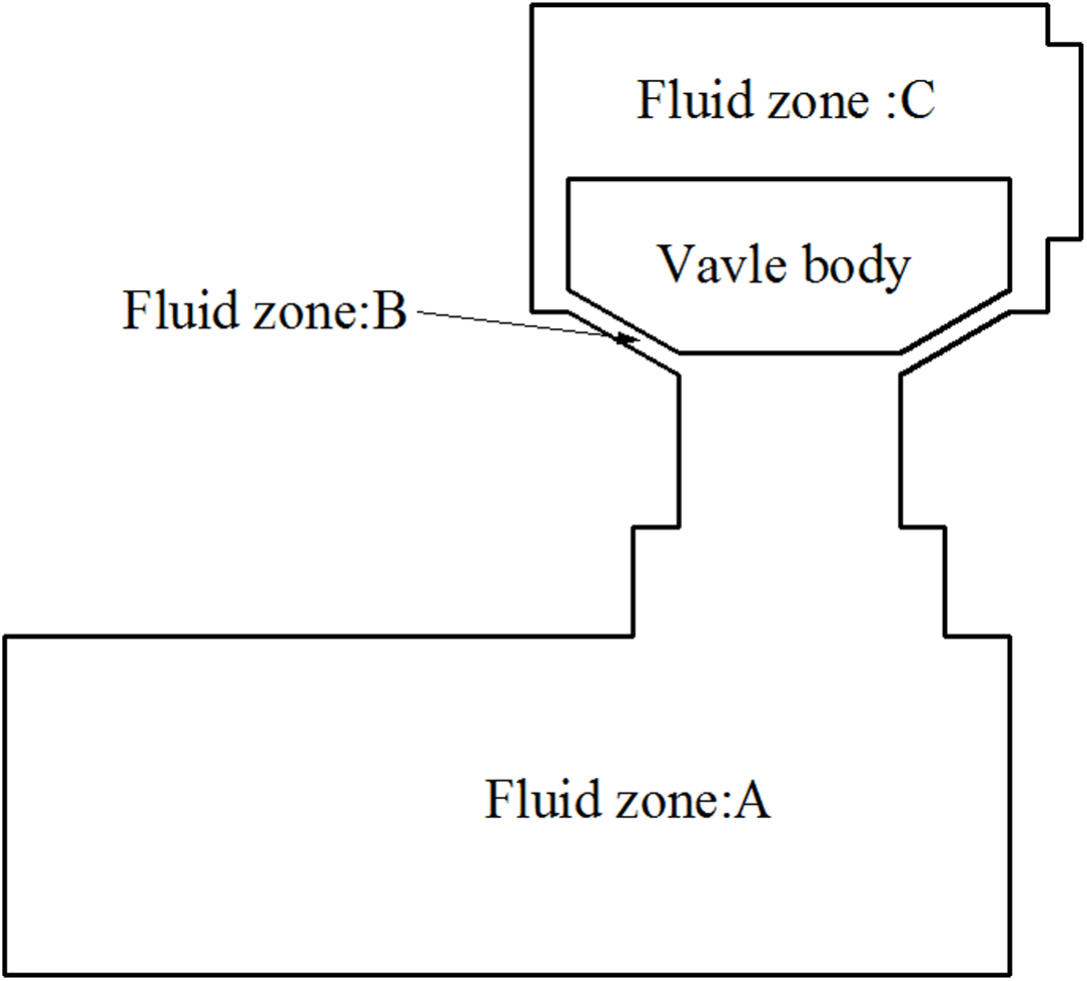
Schematic showing the division of fluid zones required for dynamic mesh motion.

The structural field model of the discharge valve body was established by using ANDINA commercial software in conjunction with the fluid-structure interaction analysis; the interaction between the valve body and the fluid was achieved by selecting the FSI pattern for analysis. The valve body is represented by a simplified plane model, because the actual reciprocating pump valve body is discoid and conical in shape. The fluid field of valve motion is divided into three zones, which is convenient for the meshing and coupling analysis of the fluid-solid interface (Fig 4). According to the literature [19], in order to reduce the level of complexity of the solution of a 3D problem in axisymmetric structure, it can translate the 3D problem to a 2D problem by setting correction factors. The plunger chamber, valve body, and discharge chamber are all symmetric geometric cylinders, allowing the fluid-structure interaction model to be simplified into a 2D plane model.

#### 2.2.2 Boundary conditions and meshing

**Fig 5:**
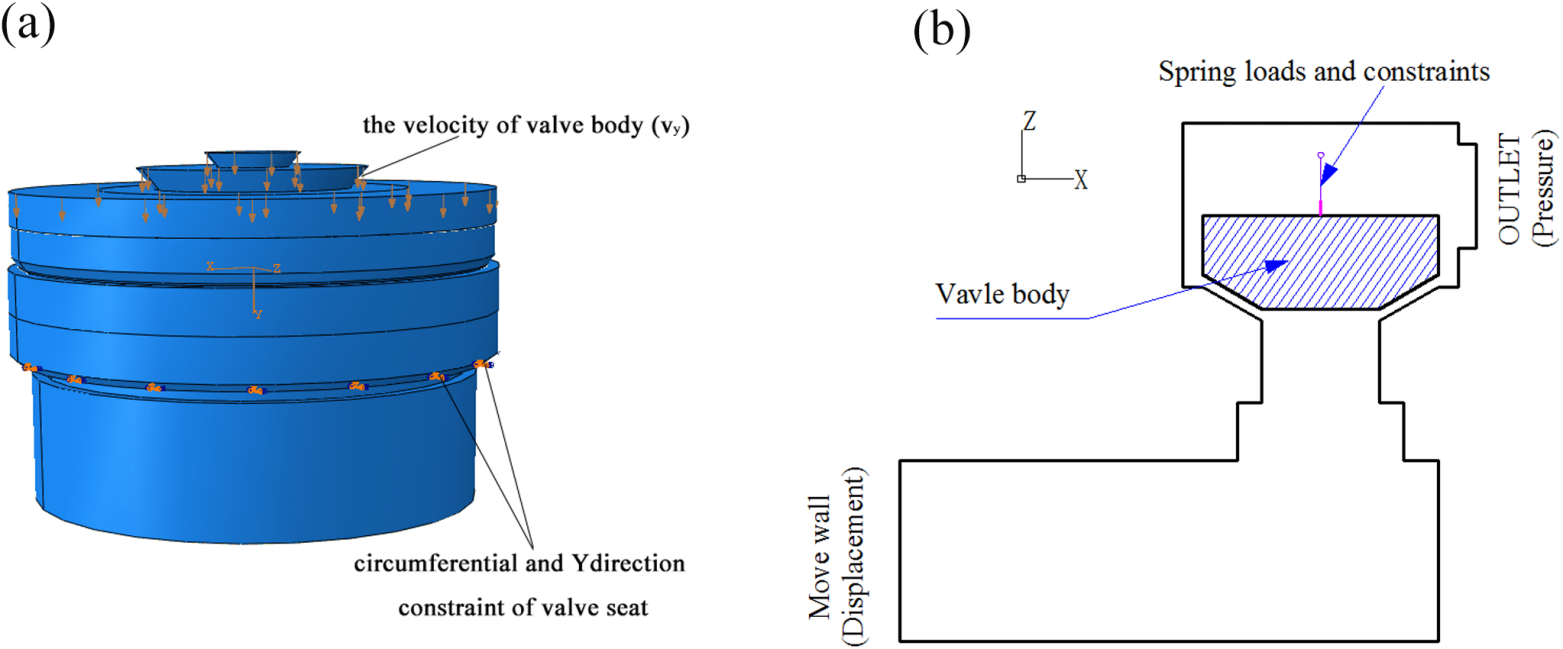
Computational regional boundary constraints. a) Valve components; b) fluid-structure interaction.

**Fig 6:**
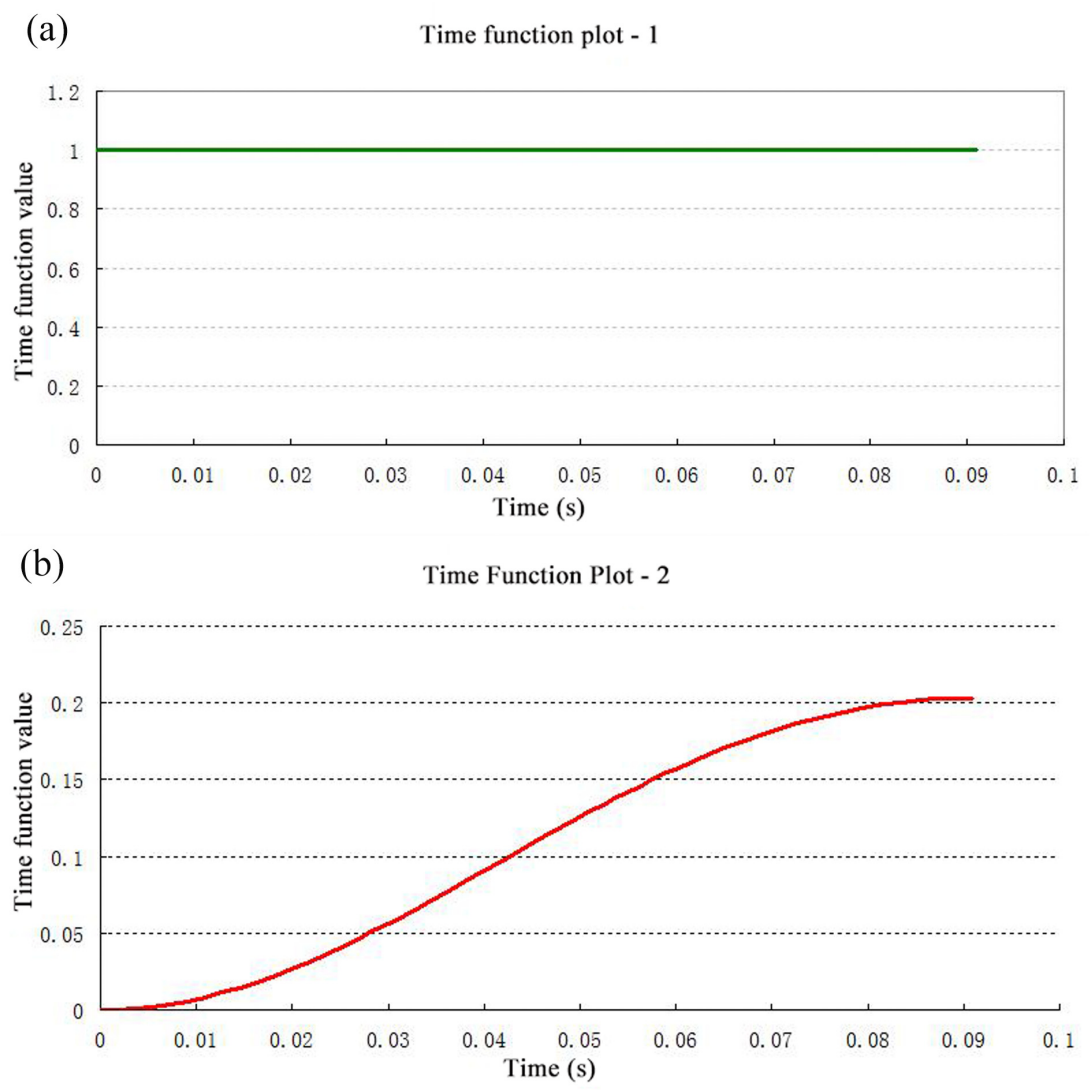
Time function for pump valve cycle. a) Time function definition for loading forces; b) time function definition for moving wall displacement.

As shown in Fig 5(A), the valve body reciprocates only in the Y direction; therefore other constraints are imposed on the valve body in addition to the y direction. Fig 5(A) illustrates the constraints imposed upon the valve body and valve seat. The large displacement hypothesis was proposed for valve motion, while the conical outer edge of the valve body is defined as a surface of fluid-structure interaction. Additionally, the load constraints on the valve body (i.e., gravity, spring pre-load and variable load) were defined by the time function plot-1, demonstrated by Fig 6(A). The spring pre-load is coupled at the center point of the upper surface of the valve body, as shown in Fig 5(B); this is completed by setting the installation distance of the connecting parts of the spring. The spring load was then applied through the valve motion; gravity is automatically loaded by inputting the density and directions of valve acceleration into the ANDINA software.

According to the working principles of the hydraulic end of the plunger pump, the plunger displacement is a sinusoidal function which varies with time; therefore the entire displacement of the moving wall is a sinusoidal function which varies with time, and is defined by time function 2, depicted in Fig 6(B). During the operation of the reciprocating pump, a pressure of 40 MPa is always present in the discharge line; therefore, the outlet of the fluid field represents the pressure boundary condition of 40 MPa, which is defined by time function 1. In addition, the other boundary conditions of the fluid field was defined as a no-slip wall boundary. The relationship between fluid flow, velocity, sectional area and pressure difference can be expressed as follow:

Q=μ*A*(2*ΔP/ρ)^0.5 (9)

Where Q is flow; μ is flow coefficient; A is cross sectional area; ∆P is the pre and post pressure difference of the pump valve; ρ is the fluid density.

Under the promotion of the plunger, the internal pressure of the liquid in the pump chamber gradually increases, and then the liquid discharges when the discharge valve opens. The variable displacement of flow field at the inlet results in the changes of the pre-and post pressure difference of the pump valve. And other parameters of Eq 9 are constant, so the discharge flows of a plunger pump continuous change.

**Fig 7:**
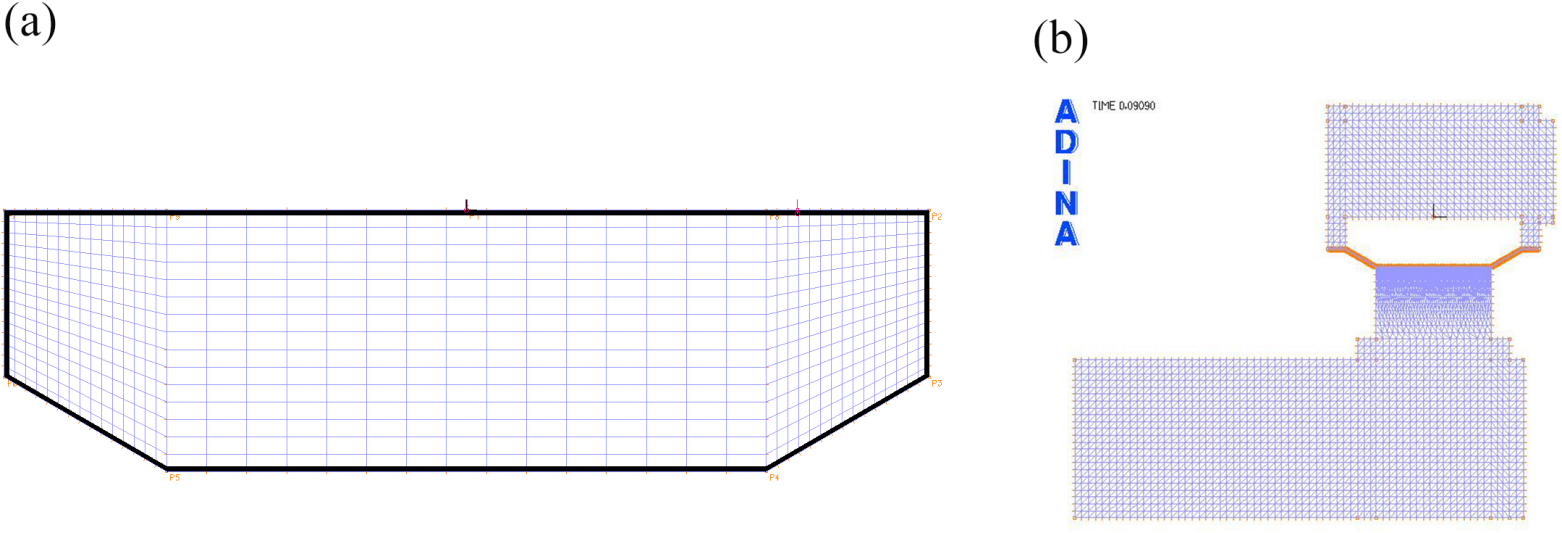
Meshing of valve body and fluid zones. a) Mesh of valve body; b) Mesh of divided fluid zones.

The mesh field of the valve body structure is shown in Fig 7(A). Mesh desity was assumed to be of fixed length, because the valve body is driven by the fluid field and the stress does not vary greatly. The mesh of the divided fluid zone is shown in Fig 7(B), consisting of mesh in the slip-wall and on the fluid-structure interaction surface. The mesh must be as fine as possible, and sections on the trajectory must demonstrate no mutations.

**Fig 8:**
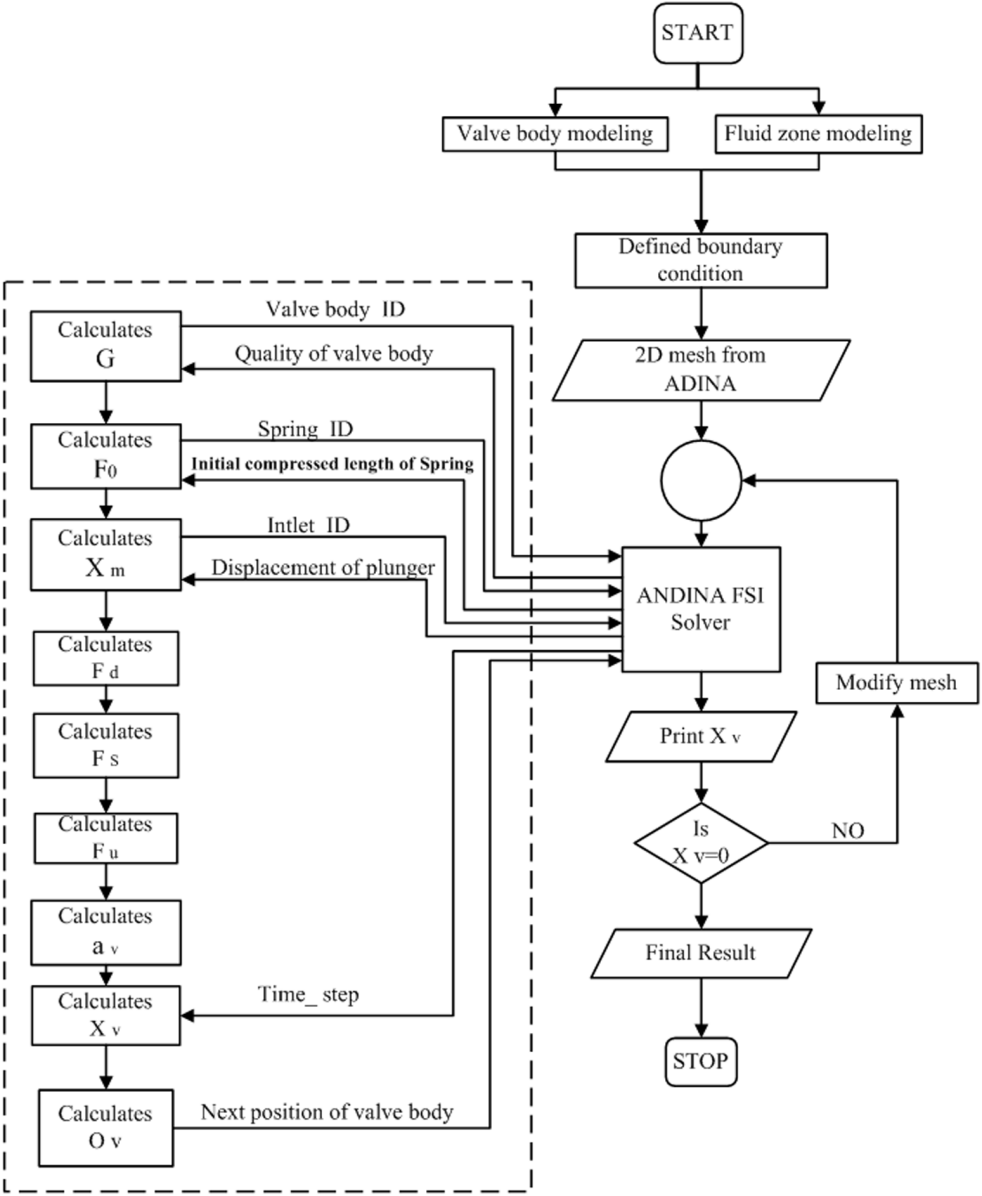
Flow chart of valve motion analysis of a reciprocating pump.

Fig 8 presents a flowchart of the finite element simulation procedure for the ANDINA iterative solution to the valve motion analysis, as described in Eq (6). First, the structure and flow model determined by the valve body dynamic analysis were established under the FSI pattern in the ANDINA software. Next, the boundary conditions were defined by the actual operating conditions of the pump valve. Then, the initial 2D mesh was introduced into the fluid and structure field domain, as shown in Fig 7. Mesh size of the valve clearance fluid field has great influence over the motion of the valve body, while the number of grid cells depends upon the valve body lift. The ALE method was employed for dynamic analysis of the mesh [20, 21]. According to this method, grid points in the fluid were continuously updating depending on the movement of the free fluid surface and the fluid-solid interface. “C” calculates the valve body displacement after calculating the various forces acting on the valve. During the calculation, the function dynamically interacts with the ANDINA FSI solver before communicating the current valve position to the solver. The mesh of the geometry needed to modify during operation of the reciprocating plunger movement was then automatically generated in the ANDINA FSI, and the flow is again analyzed for the new geometry. This cycle of calculation continues until the valve body comes to a full stop.

## Experimental Design of Valve Motion

### 3.1 Test principle and devices

**Fig 9:**
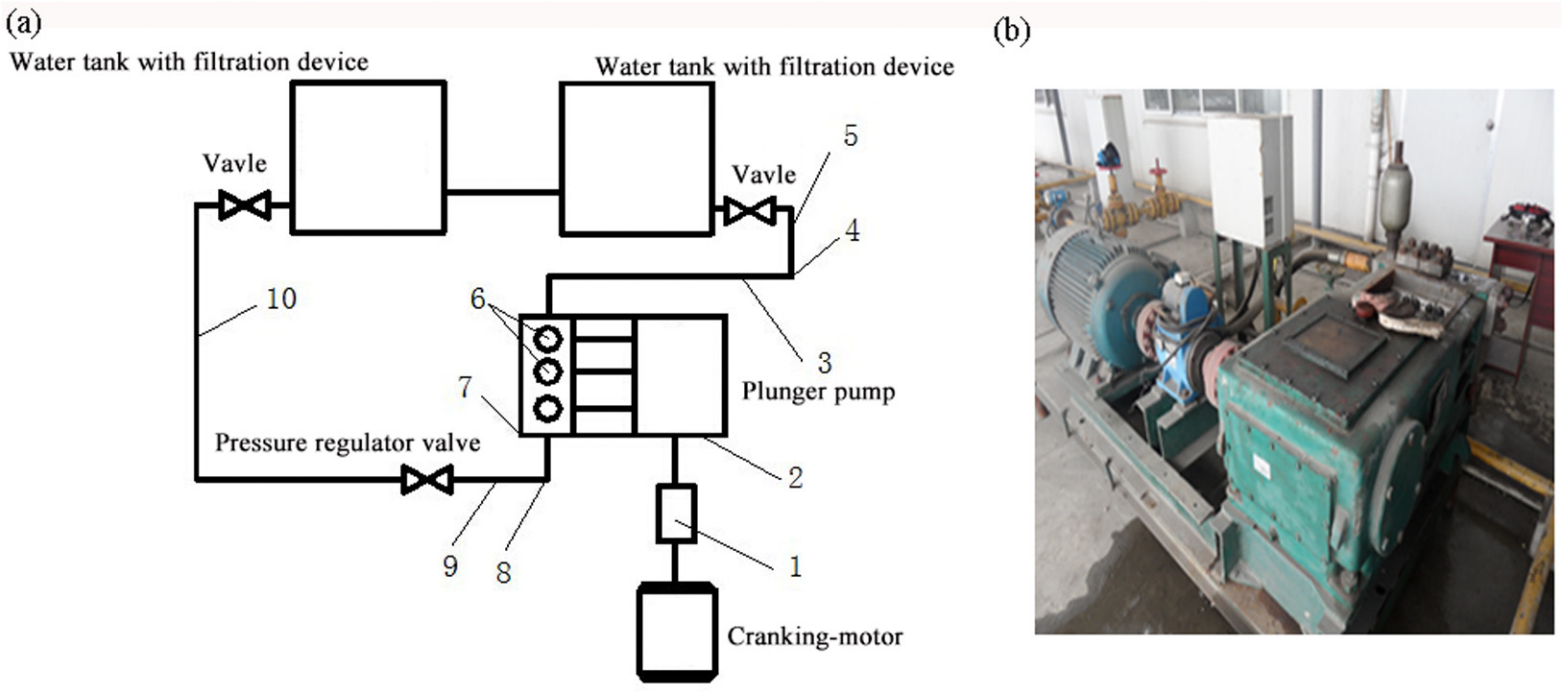
Test principle diagram of valve motion. 1) Torque speed sensor; 2) angular displacement sensor; 3) inhalation flow sensor; 4) temperature sensor; 5) pressure sensor; 6) displacement sensor; 7) vibration sensor; 8) discharge pressure sensor; 9) discharge flow sensor; 10) temperature sensor.

A 3DS-7/12.5 triplex plunger pump was employed for experiment (S1 Fig). It was driven by power plant (including a variable frequency motor and torque speed sensors, etc.) in order to pump the water medium into the piping system (S2 Fig), which would then flow back to the water tank, thus forming a closed system. The plunger displacement sensor is installed in the power end of the plunger and the discharge valve displacement sensor is installed in the valve body of the discharge valve (S3 Fig). In this process, data from various sensors was automatically collected via a data acquisition card (Altai Beijing science and Technology Co., Ltd.USB2821) (S4 Fig); data was then saved and displayed in the computer (S5 Fig), which was shown in Fig 9.

The used experimental devices mainly consisted of three parts: machinery equipment, electrical equipment and a data acquisition system. The machinery equipment was composed of a 3DS-7/12.5triplex plunger pump, a Y250M-6 motor with a frequency converter, a fluid reservoir, as well as discharge and suction piping lines and valves. The electrical equipment comprises a torque and speed sensor, split differential voltage linear displacement sensor (made in Shenzhen Si Ming Wei Testing Equipment Co., Ltd.), a pump body vibration sensor, a flow sensor (made in Tianjin Si Mite Precision Instrument Co., Ltd.), and a switch control cabinet. The data acquisition system included a data acquisition card (made at Beijing Altai Science and Technology Development Co., Ltd.), as well as transmission cables and a computer.

### 3.2 Experimental conditions

This experiment was conducted at room temperature with a water test medium. The motor speed was 250 rpm, with a calibration discharge pressure of 5 MPA. The valve body mass and spring stiffness are depicted in Table 2.

**Table 2:** Experimental valve quality and spring stiffness.

m_1_(g)	m_2_(g)	C_1_(N/mm)	C_2_(N/mm)	C_3_(N/mm)	C_4_(N/mm)
156	85	3.2	6.1	10.2	18.5

C, spring stiffness; m, the quality of valve body.

### 3.3 Acquisition and processing of test data

**Fig 10:**
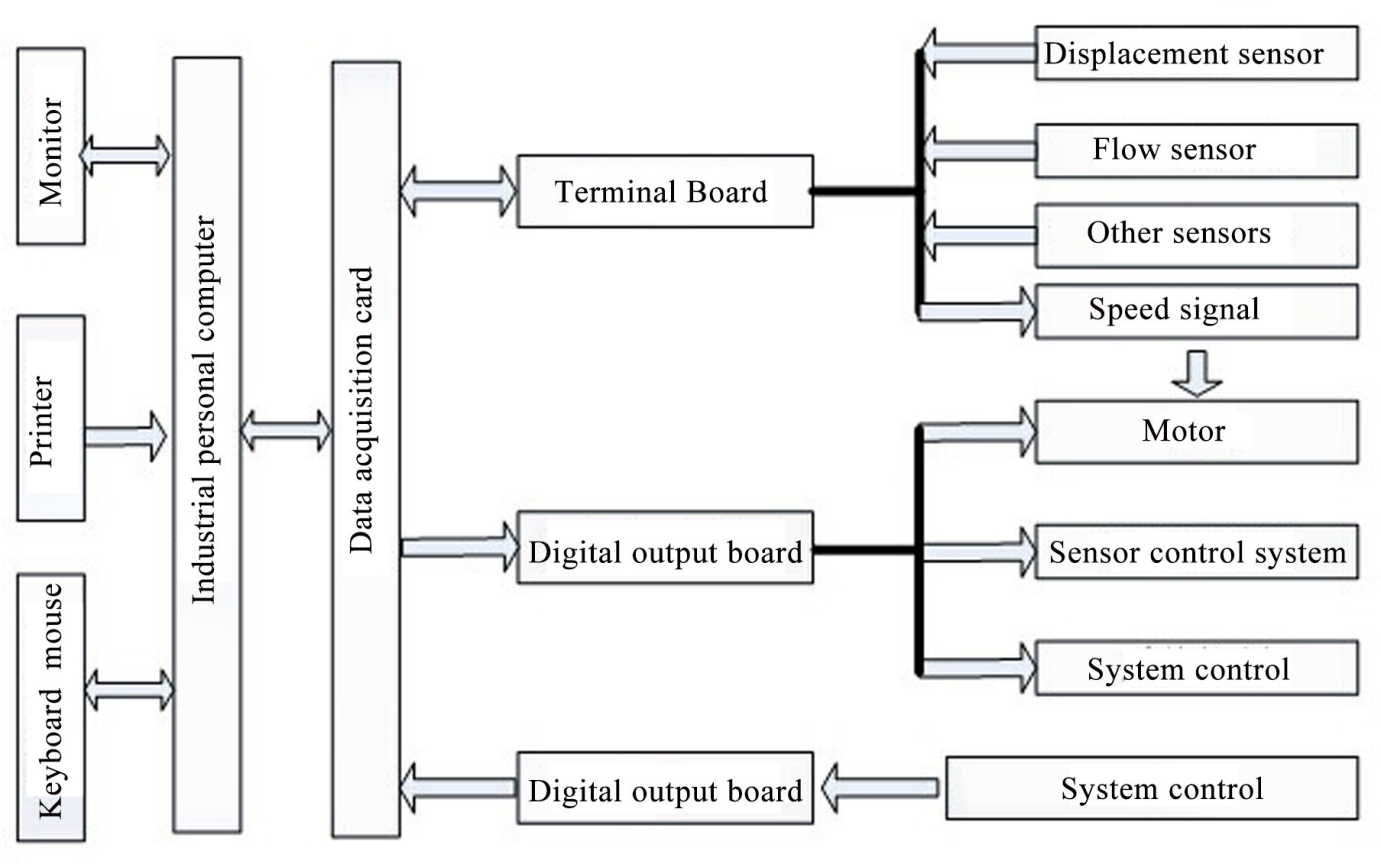
Data acquisition system schematic diagram.

Fig 10 depicts the data acquisition system diagram. All signals collected by sensors were inputted to the data acquisition card, and the motor speed was adjusted by a system control signal. Calibration of sensors (S1 Table) and collection frequency were determined before data collection. According to the highest frequency of pump valve motion, the collection frequency should meet the accuracy of the valve displacement (900Hz).during the discharge valve motion cycle, requiring more than 100 collected data points (S1 Text).

## Results and Discussion

### 4.1 Effects of spring stiffness on valve motion

**Fig 11:**
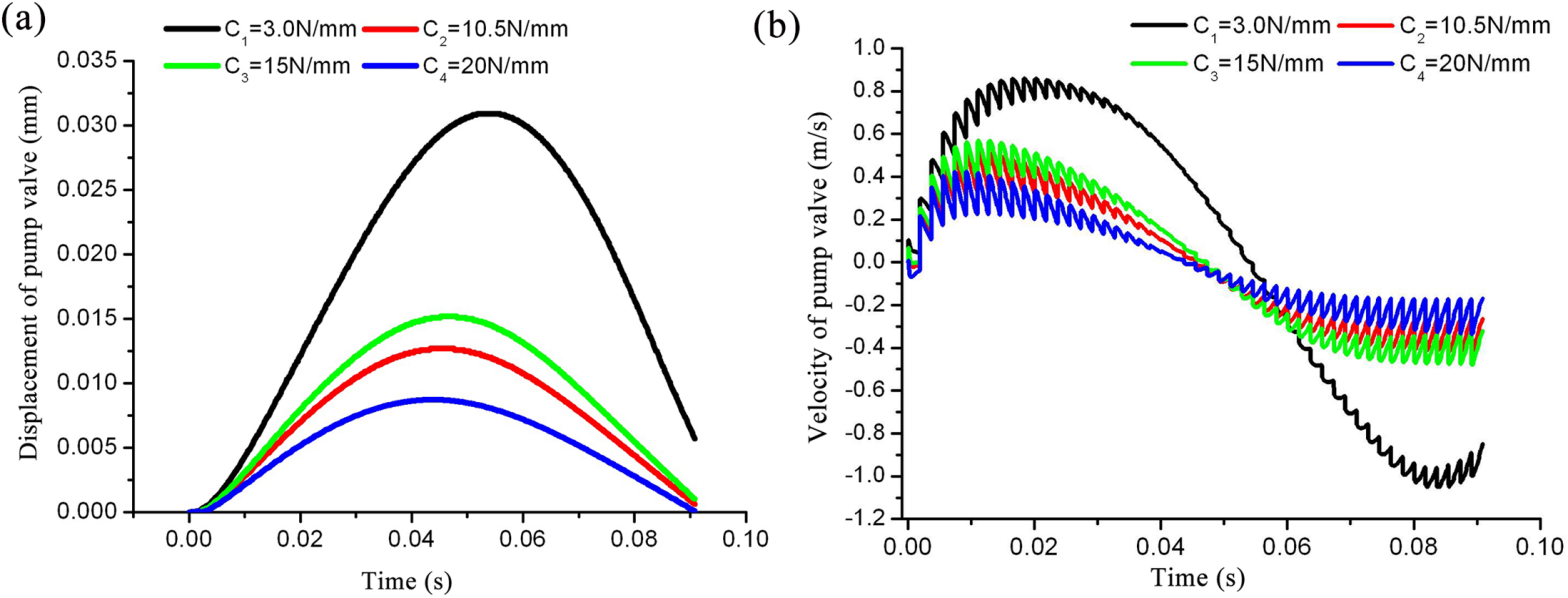
Curve of valve motion as a function of time with different spring stiffnesses. a) Displacement curve comparison; b) velocity curve comparison.

Valve lift and velocity reach their respective maxima when the spring stiffness is equal to C_1_, indicating the smallest valve opening resistance. Alternatively, the valve lift and velocity reach their respective minima when the spring stiffness is equal to C_4_, indicating that the opening valve pressure is the highest. The maximum lift of the valve is reduced accordingly with an increase in spring stiffness; when the plunger moves to its terminal point, the valve retains its distance to the valve seat. Moreover, lower spring stiffness results in smaller valve body opening resistances, as shown in Fig 11; however, this also results in a larger shut-off lag angle, as depicted in Fig 11(A) and 11(B). Therefore, in order to optimize the dynamic performance of the valve, the optimized design in spring stiffness must be investigated and realized.

### 4.2 Effects of the valve mass on valve motion

**Fig 12:**
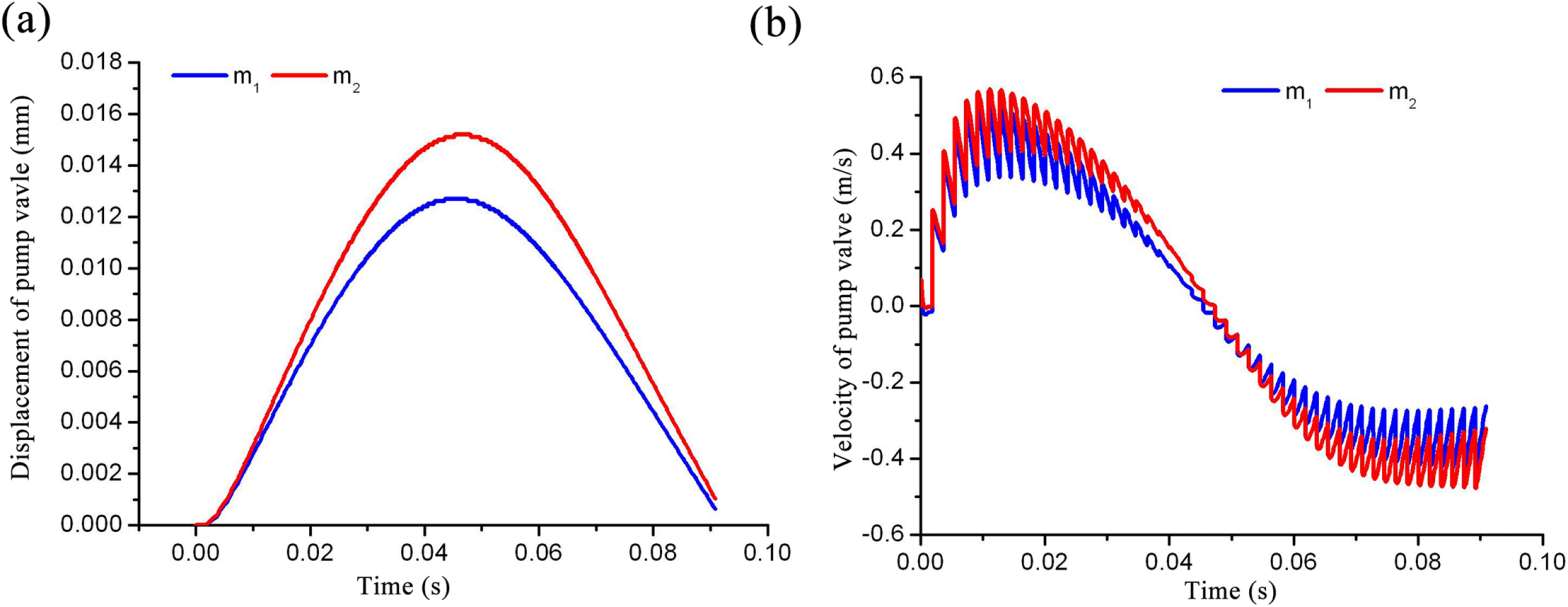
Curve of valve motion as a function of time with different valve masses. a) Displacement curve comparison; (b) velocity curve comparison.

When the spring stiffness is fixed at 10.05N/mm, the quality of the valve body is altered by the density of the valve. Assuming a density of 3.8kg/cm^3^ and an original valve quality of m_1,_ when the density is reduced to half then the quality of the valve is denoted as m_2_. Valve quality affects the lift and speed of the valve, as shown in Fig 12, and a reduction in valve quality results in increases to both the maximum displacement and speed of the valve. Thus, the valve quality parameter has an effect on the opening resistance and shut-off lag angle of the valve, but has little effect on valve movement relative to the effect induced by spring stiffness, as shown in Fig 12.

### 4.3 Effects of spring stiffness and valve quality on fluid velocity

**Fig 13:**
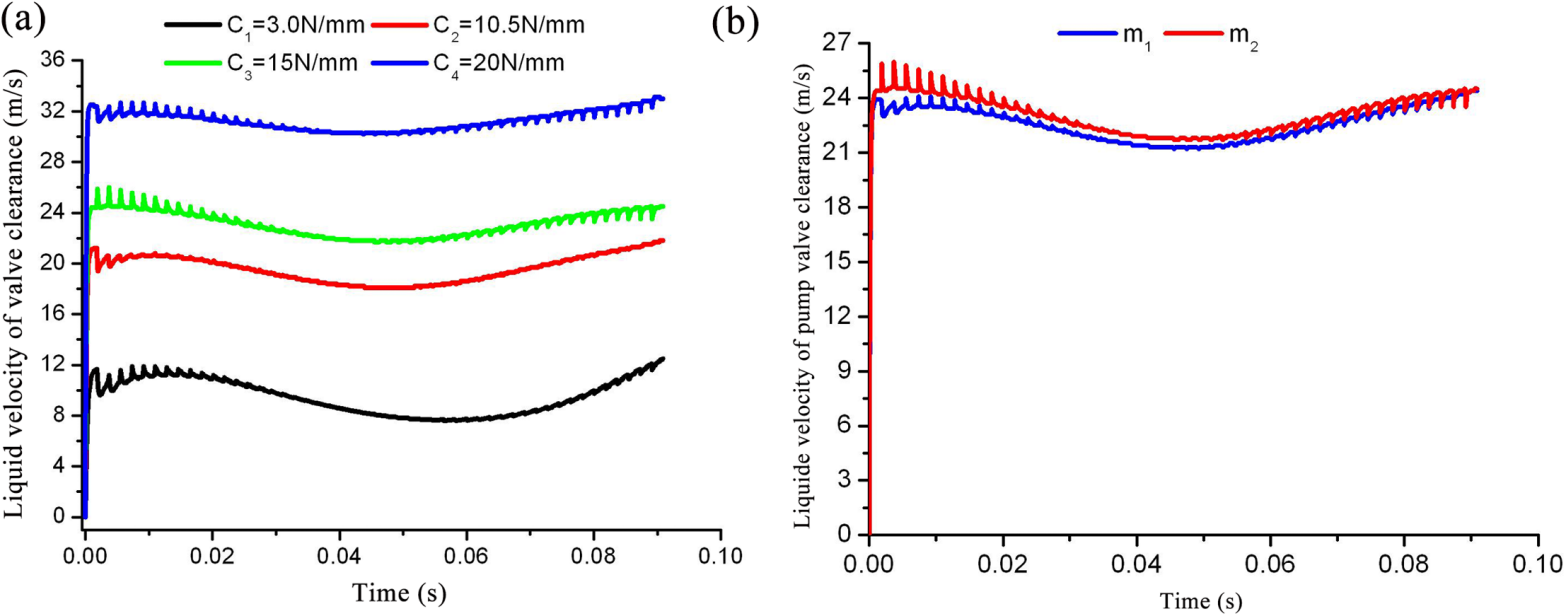
Curve of fluid velocity of valve clearance as a function of time. a) Fluid velocity curves of different spring stiffnesses; b) fluid velocity curve of different valve masses.

Flow field analysis of valve motion does not only obtain the erosion position and eddy current situation, but also has the potential to improve the hydraulic end structure of the reciprocating pump. The clearance between the valve and the valve seat changes with time, therefore, the liquid velocity analysis for valve clearance significantly impacts structural and kinematics optimization (S1 Movie). Greater sprint stiffness correlates to smaller flow areas of valve clearance. According to the law of continuous flow mass conservation, the velocity of the fluid which flows through the valve clearance is larger under identical plunger speed conditions, as shown in Fig 13(A). Higher fluid velocities tend not only to form vortices, which result in energy loss, but often the flow speed is so great as to induce gasket seal leakage and the deformation of erosion rubber; the rubber damage is particularly significant when the fluid medium is intermixed with hard particles. This is also consistent with more than 80% of observed valve failures caused by rubber erosion in oil fields. As shown in Fig 13(B), variations in valve quality have particular impact upon valve motion; however, the effect is small in comparison to the effects induced by the spring stiffness parameter.

**Fig 14:**
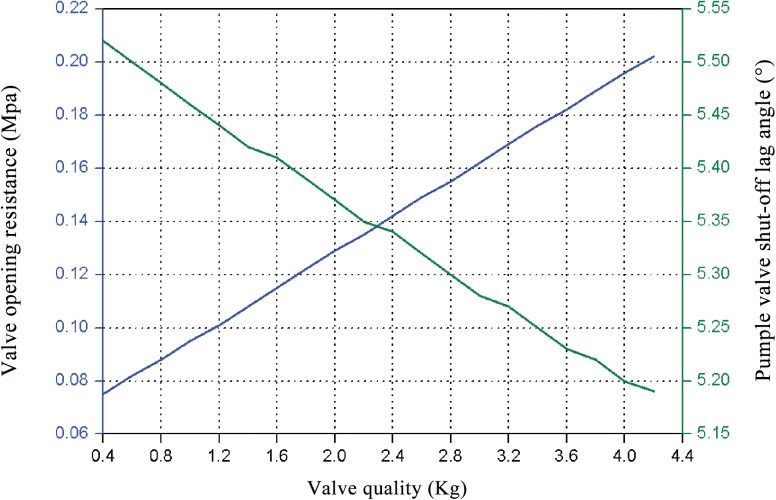
Curve of valve opening resistance and shut-off lag angle with respect to valve quality.

Valve quality has little effect on the shut-off lag angle, due to the smaller effect of gravity when compared to the spring force. However, increasing valve quality correlates to greater opening resistances, as shown in Fig 14. Therefore, there is an optimal parameter value of valve quality in order to obtain better valve motion performance. The optimized valve quality is 2.3Kg, which produces a volume efficiency increase of 0.04d when compared to the original valve quality. Therefore, the impact of pump valve quality on volume efficiency may be negligible, but valve quality is closely related to the stable operation of the pump valve.

**Fig 15:**
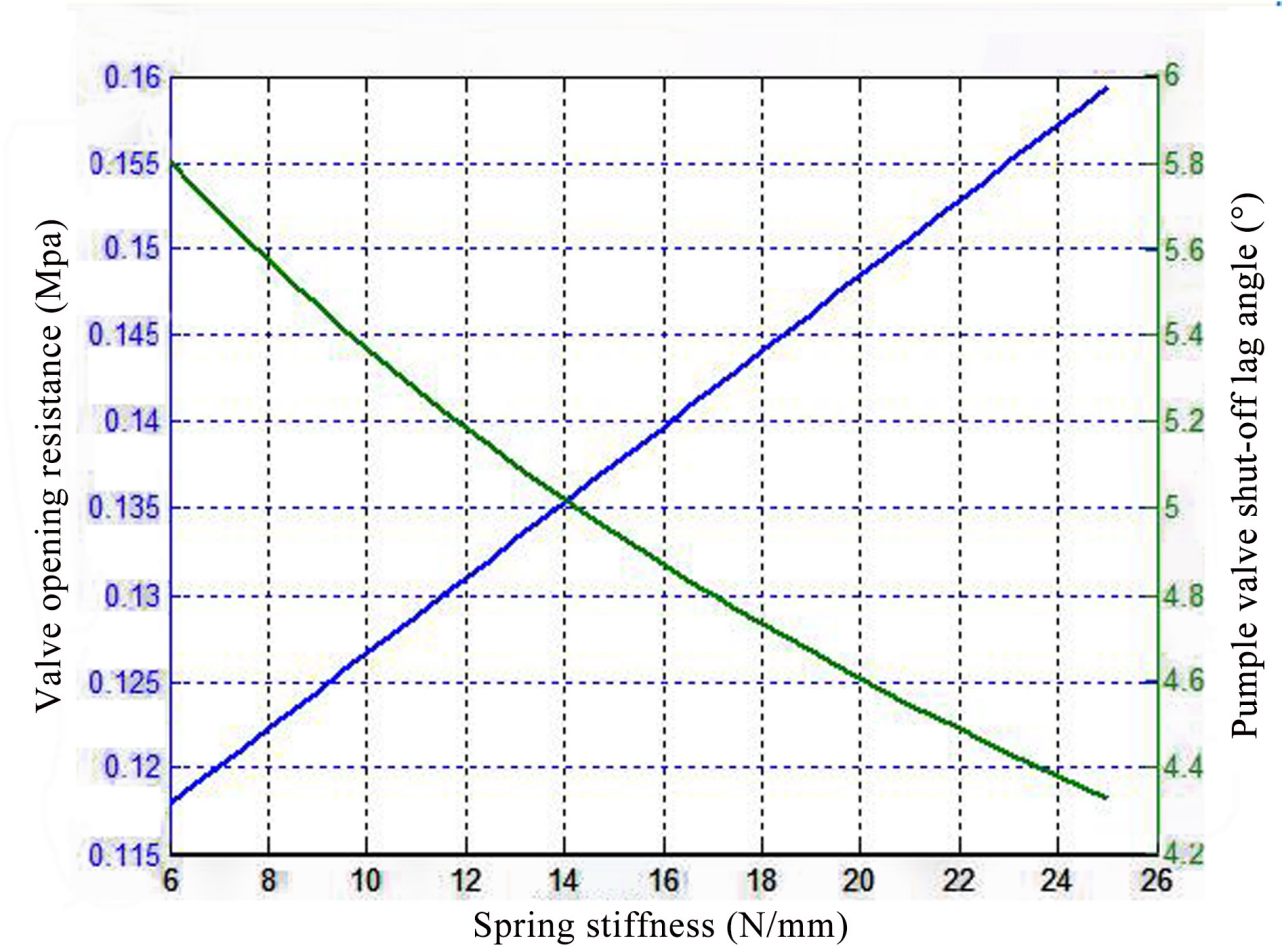
Curve of valve opening resistance and shut-off lag angle with respect to spring stiffness.

The optimized spring stiffness was obtained by investigating the influence of various stiffness values on shut-off lag angle and the opening resistance of the valve, as shown in Fig 15. As the spring stiffness increases, the opening resistance gradually increases and the shut-off lag angle is gradually reduced. Therefore, the intersection between the two variables provides the optimal spring stiffness for a 2000-fracturing pump valve: 14.6N/mm. The volume efficiency of the pump valve increased by 4‰, as compared to the volume efficiency achieved with the original spring stiffness of 10.09N/mm.

### 4.4 Effects of spring stiffness on impact contact of valve

**Fig 16:**
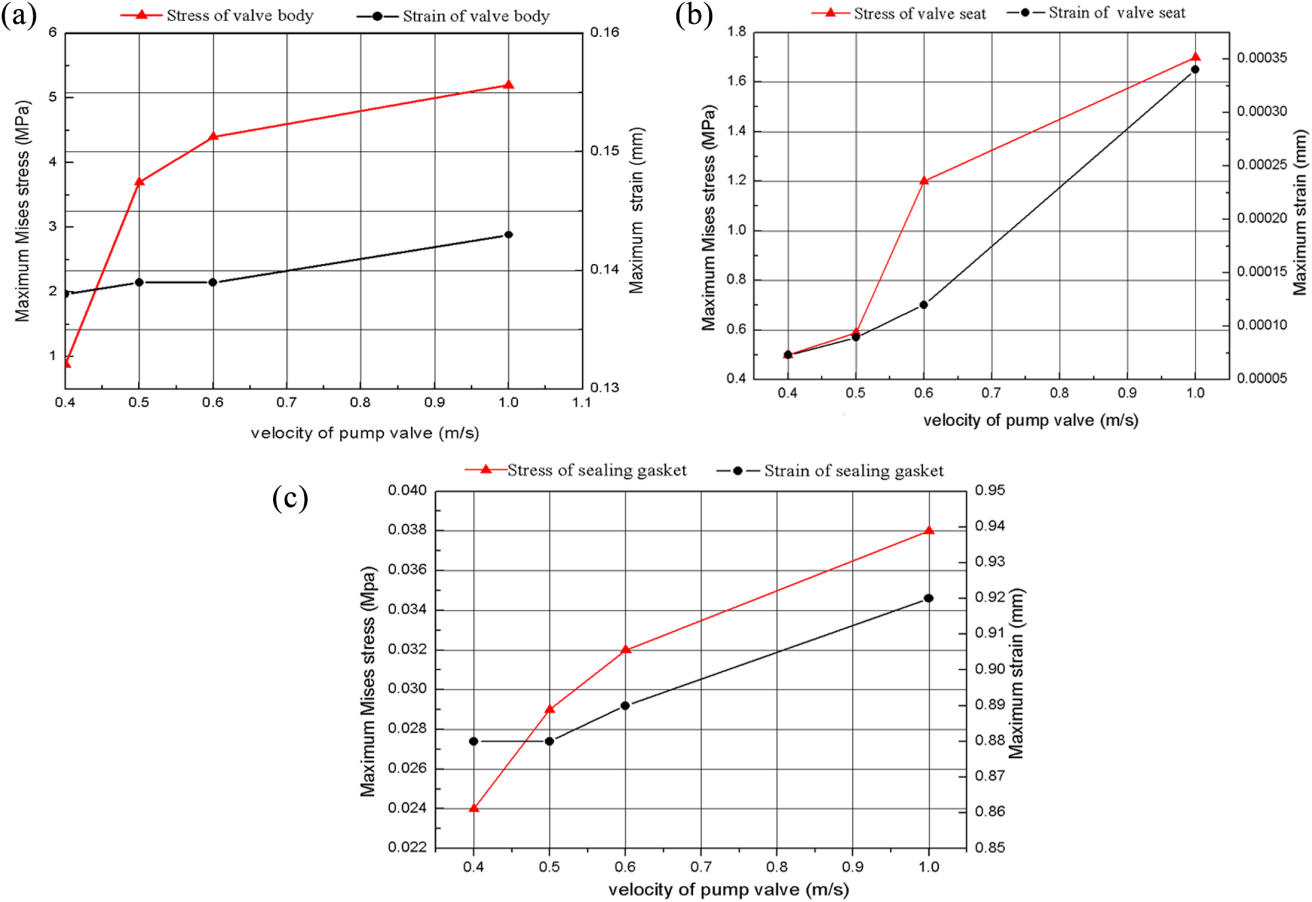
Stress and strain curve of valve components with pump valve velocity under impact contact. a) Valve body; b) valve seat; c) sealing gasket.

Dynamics analysis of the discharge valve motion demonstrated that impact contact occurred during the reciprocating movement between the valve body and the valve seat, and that impact contact was the primary cause of damage to the valve assembly. Therefore, contact analysis of valve assembly was conducted for various pump valve spring stiffness values. Valve assemblies include the valve body, a sealing gasket and the valve seat; the material of the valve body and seat is 20CrMnTi, while the sealing gasket is made of polyurethane rubber. The boundary conditions and constrains of valve impact contact analysis are shown in Fig 5(B). According to the actual operating conditions of the hydraulic end of the plunger pump, the seating velocity of the valve body with various spring stiffness values is 1m/s, 0.6m/s,0.5m/s and 0.4m/s. The maximum stress-strain curve of different pump valve seating velocities is shown in Fig 16, based on ABQUS finite element analysis (S6 Fig). Results indicate that the impact contact stress and strain of the valve body and valve seat increase with an increase in seating velocity; however, the increased amplitude is small, and far less significant that the yield stress of the material. Therefore, variation in the pump valve seating velocity has relatively small influence on the service life of the valve assembly. However, the fluid-solid interaction and impact contact analysis of the hydraulic end of the pump demonstrate that greater spring stiffness of the valve produces higher liquid velocities of valve clearance, and subsequently greater impact contact stress and strain of the valve assembly. Finally, the excessive liquid velocity of the valve clearance caused erosion and washout damage to the sealing gasket.

The reciprocating plunger pump is mainly used in the fracturing process in oil exploration. Therefore, the volumetric efficiency of the pump valve is closely related to the working performance of the fracturing pump. Moreover, the opening lag angle of the discharge valve increased with the increase of the opening resistance, which influenced the instantaneous erosion rate of the valve clearance fluid, accelerating the erosion failure of the valve components. And the shut-off lag angle was the main parameter, which influenced the volume efficiency of the pump valve (Eq 8). Figs 14 and 15 are the curves that the opening resistance and shut-off lag angle varies with different spring stiffness and valve body quality. It indicated that the two variables intersections of the pump valve opening resistance and shut-off lag angle are the optimized spring stiffness and valve body quality values. And further, the maximum stress and strain values of valve components (as shown in Fig 16) are in the range of the allowable safety when the maximum seating velocity of the valve body is 1m/s for the optimized spring stiffness and valve body parameters (S2 Table). Therefore, the optimization design can’t result in the impact and fatigue failure of valve components.

### 4.5 Experimental analysis of valve motion

**Fig 17:**
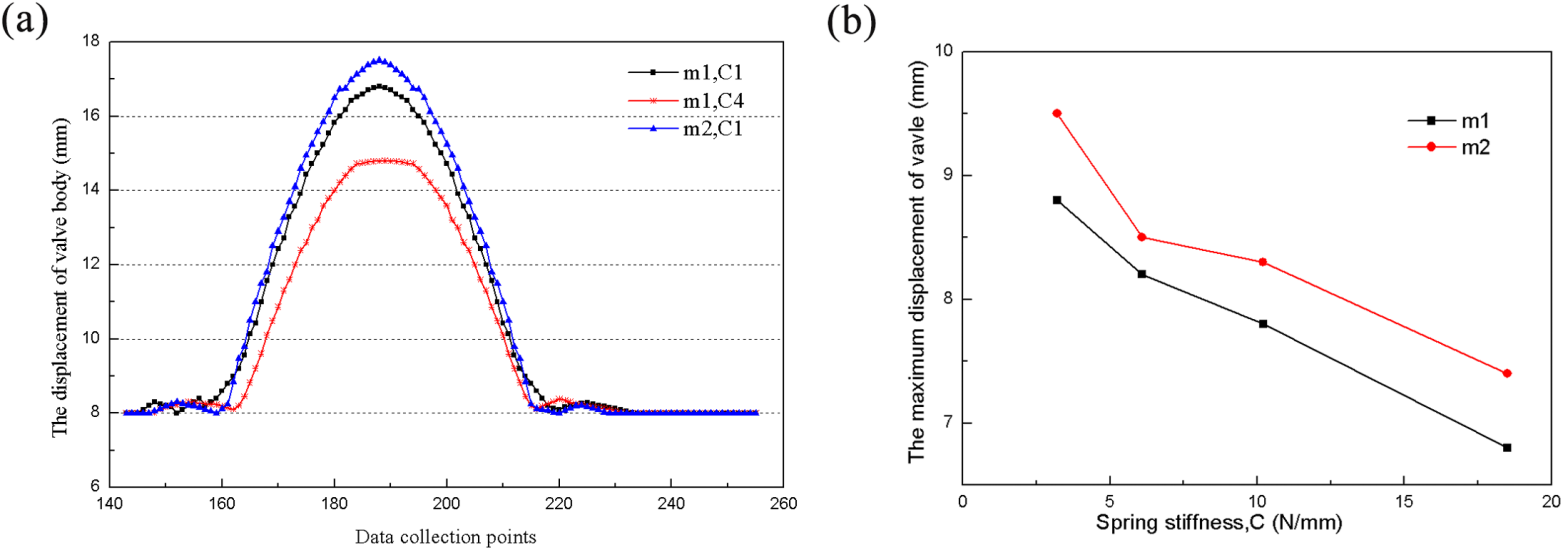
Curve of valve lift and maximum displacement under identical conditions. a) Valve lift curve with collection point; b) maximum displacement curve with respect to spring stiffness.

Under the conditions of the same valve quality and motor speed, when the spring stiffness of the pump valve is greater, the opening time of the pump valve is relatively delayed while the closing time is relatively early; additionally, the valve opening resistance increases, while the closing lag time decreases. Moreover, larger spring stiffness results in smaller valve lifts. At a fixed spring stiffness and motor speed, results indicate that lower valve quality results in large valve lifts, but little effect on opening and closing lag times, as shown in Fig 17. By comparing the experimental results of valve motion with the computer simulation results, the mathematical model and simulation calculation methods have been verified.

## Conclusion

Theoretical exploration and physical experiments were conducted to investigate the effect spring stiffness and valve quality on the opening resistance, shut-off lag angle and fluid velocity of valve clearance of valve motion for a 2000-fracturing pump under operating conditions. The kinematic behaviors of valve motion for a 2000-fracturing pump were compared and analyzed with regard to spring stiffness and valve quality on the discharge side of the pump. The following conclusions were drawn based on the obtained theoretical and experimental results:

1. A mathematical model was successfully established to describe the discharge valve motion of a 2000-fracturing pump; discrete solutions to differential equations were achieved via FSI simulation of ANDINA software.
2. FSI simulation results indicated that spring stiffness and valve quality have significant impacts on the nonlinear motion of the valve body of a 2000-fracturing pump. In comparison to the valve quality, spring stiffness demonstrated a greater impact on maximum lift, opening resistance, shut-off lag angle, the fluid velocity of clearance and the volume efficiency of the pump valve. The optimized spring stiffness was identified as 14.6N/mm; the volumetric efficiency of the pumping valve increased by 4‰ compared to results obtained with the original spring stiffness of 10.09N/mm.
3. Pump valve seating velocity has a relatively small impact on the service life of the valve assembly for various spring stiffnesses. However, greater spring stiffness results in greater flow velocity of valve clearance, which has obvious influence on erosion of the pump valve.
4. The experimental results indicated that the mathematical model and FSI simulation results correctly interpreted the effects of spring stiffness and valve quality on the valve lift and closing lag time of the 2000-fracturing pump.

## Supporting Information

S1 Fig A 3DS-7/12.5 triplex plunger pump. (TIF)

S2 Fig Torque speed sensors and frequency converter. (TIF)

S3 Fig The installation of displacement sensors. (TIF)

S4 Fig USB2821 data acquisition card. (TIF)

S5 Fig The data acquisition process. (TIF)

S6 Fig The stress and strain nephogram of discharge valve components. (TIF)

S1 Movie The fluid field distribution during the simulation of fluid structure interaction. (MPG)

S1 Table Calibration zero of displacement sensors. (DOCX)

S2 Table The stress and strain values of pump valve components under different seating velocity for the optimized spring stiffness and valve body quality. (DOCX)

S1 Text The lift of discharge valve body in a stroke under different data collection points. (TXT)
