# Supplementary figures and images for: Dynamic Behavior of Reciprocating Plunger Pump Discharge Valve Based on Fluid Structure Interaction and Experimental Analysis
Source: PLoS One. 2015 Oct 21;10(10):e0140396. doi: 10.1371/journal.pone.0140396 (PMC4619497; doi:10.1371/journal.pone.0140396)

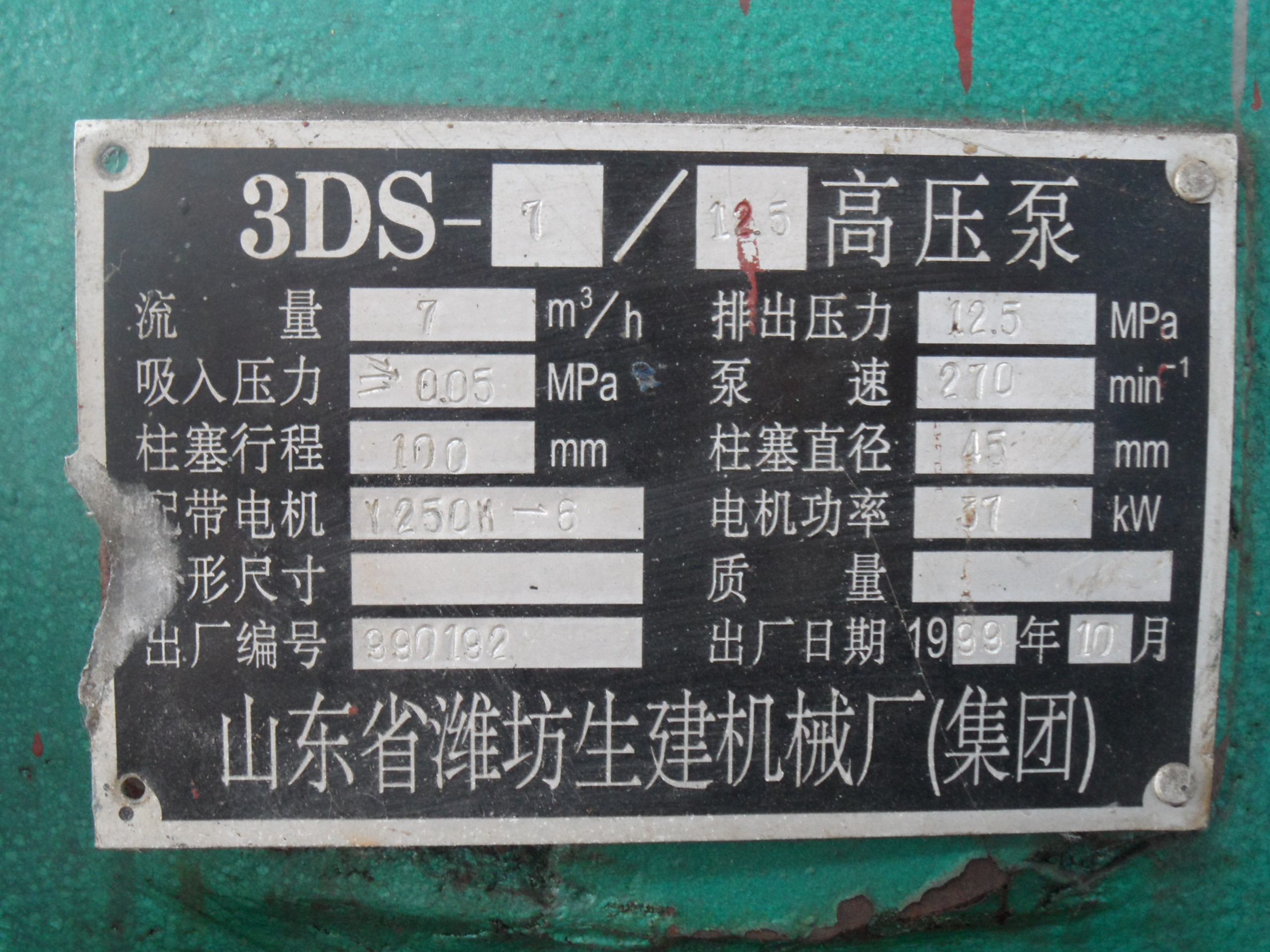

Supplement: S1 Fig — (TIF) [file pone.0140396.s001.tif]

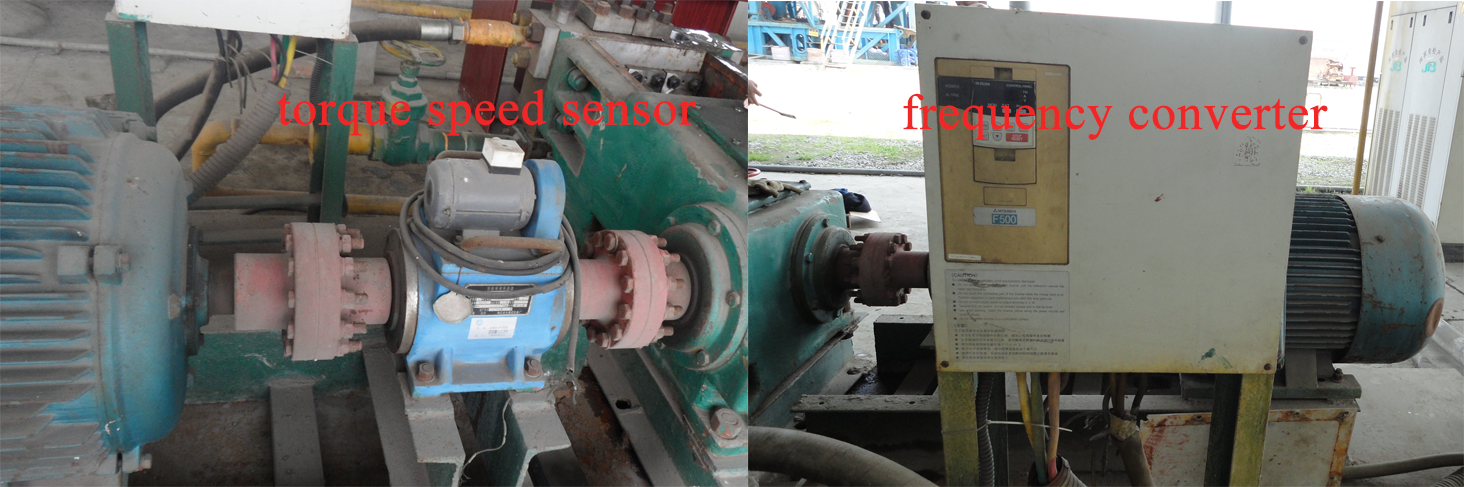

Supplement: S2 Fig — (TIF) [file pone.0140396.s002.tif]

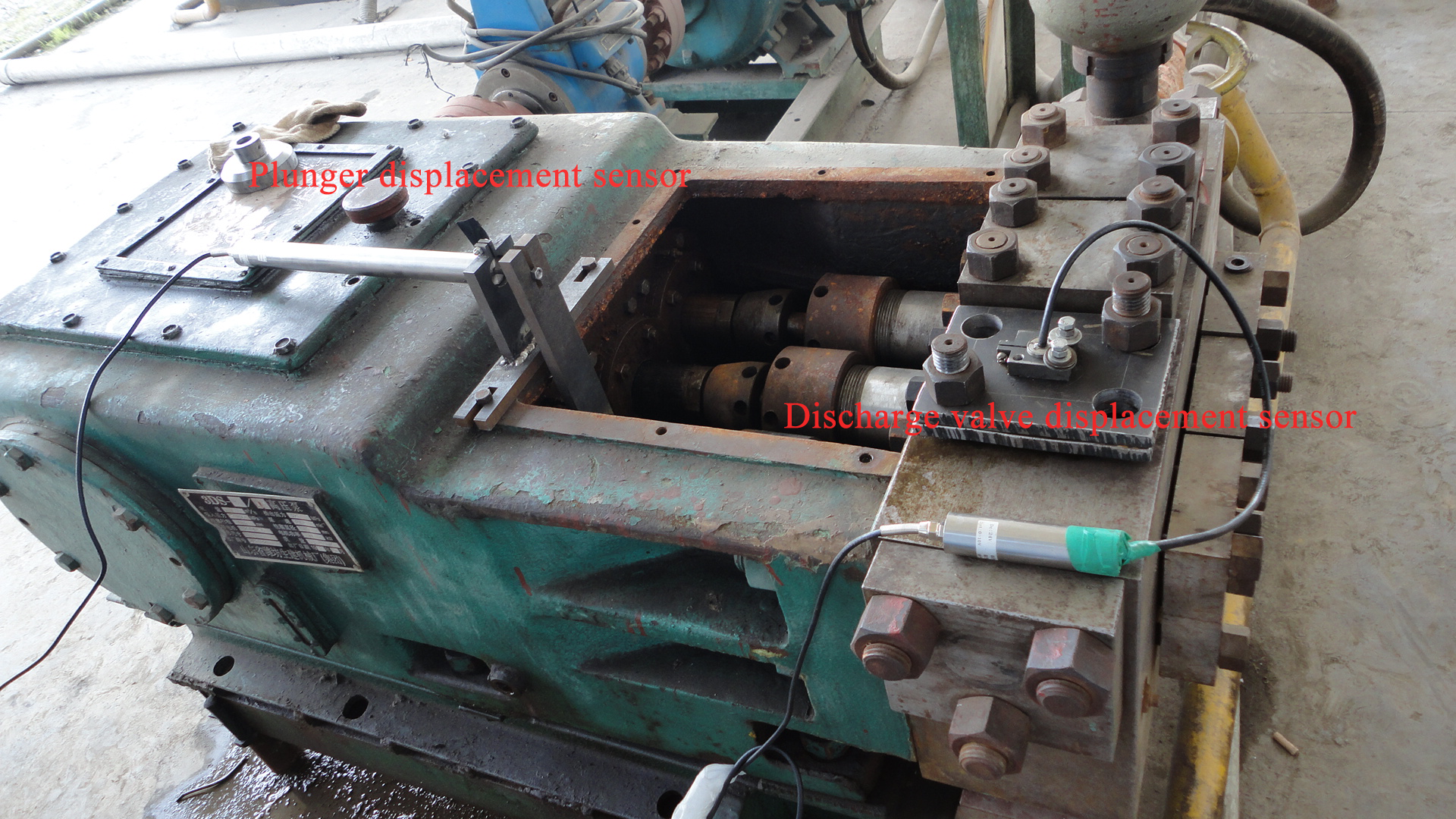

Supplement: S3 Fig — (TIF) [file pone.0140396.s003.tif]

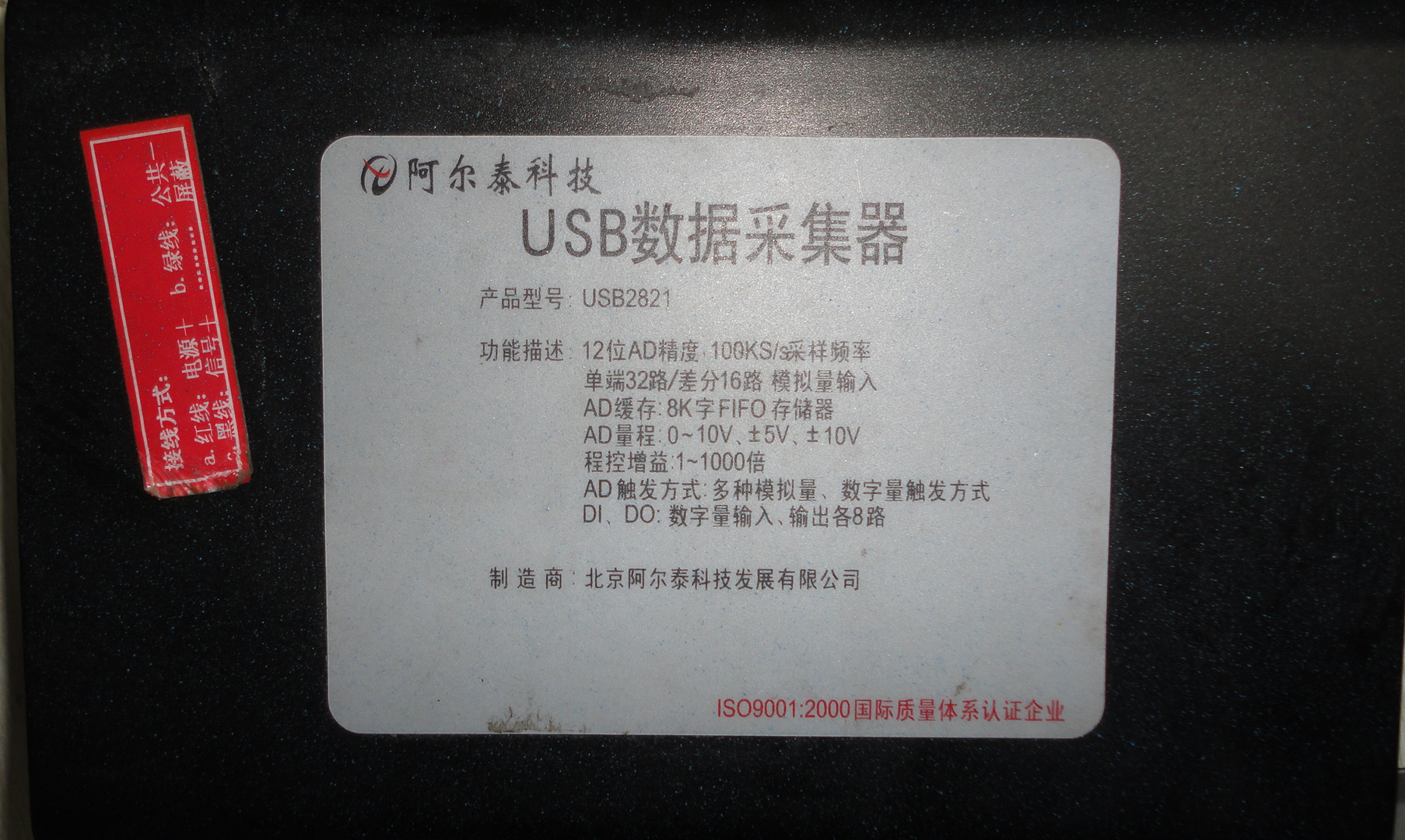

Supplement: S4 Fig — (TIF) [file pone.0140396.s004.tif]

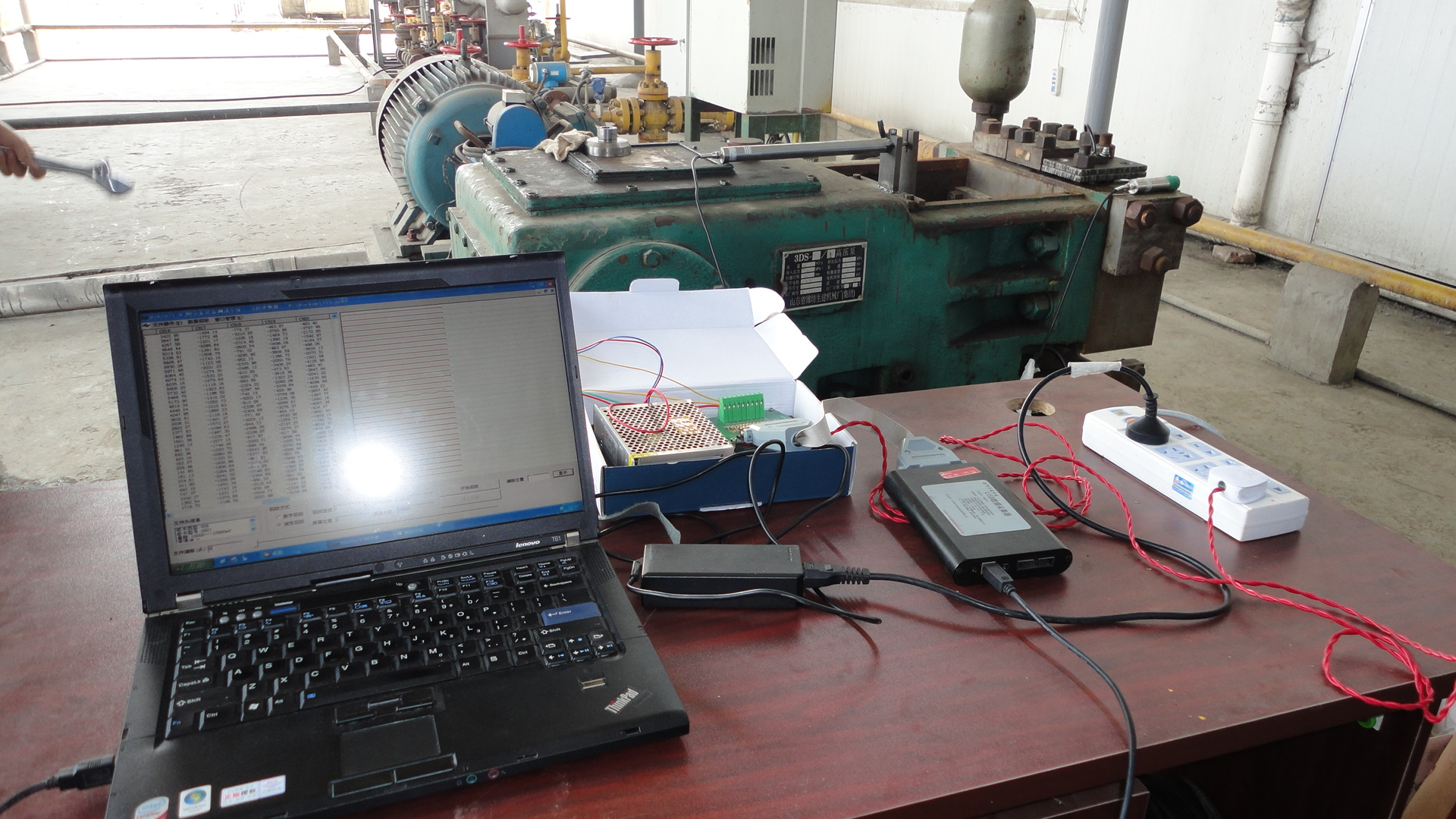

Supplement: S5 Fig — (TIF) [file pone.0140396.s005.tif]

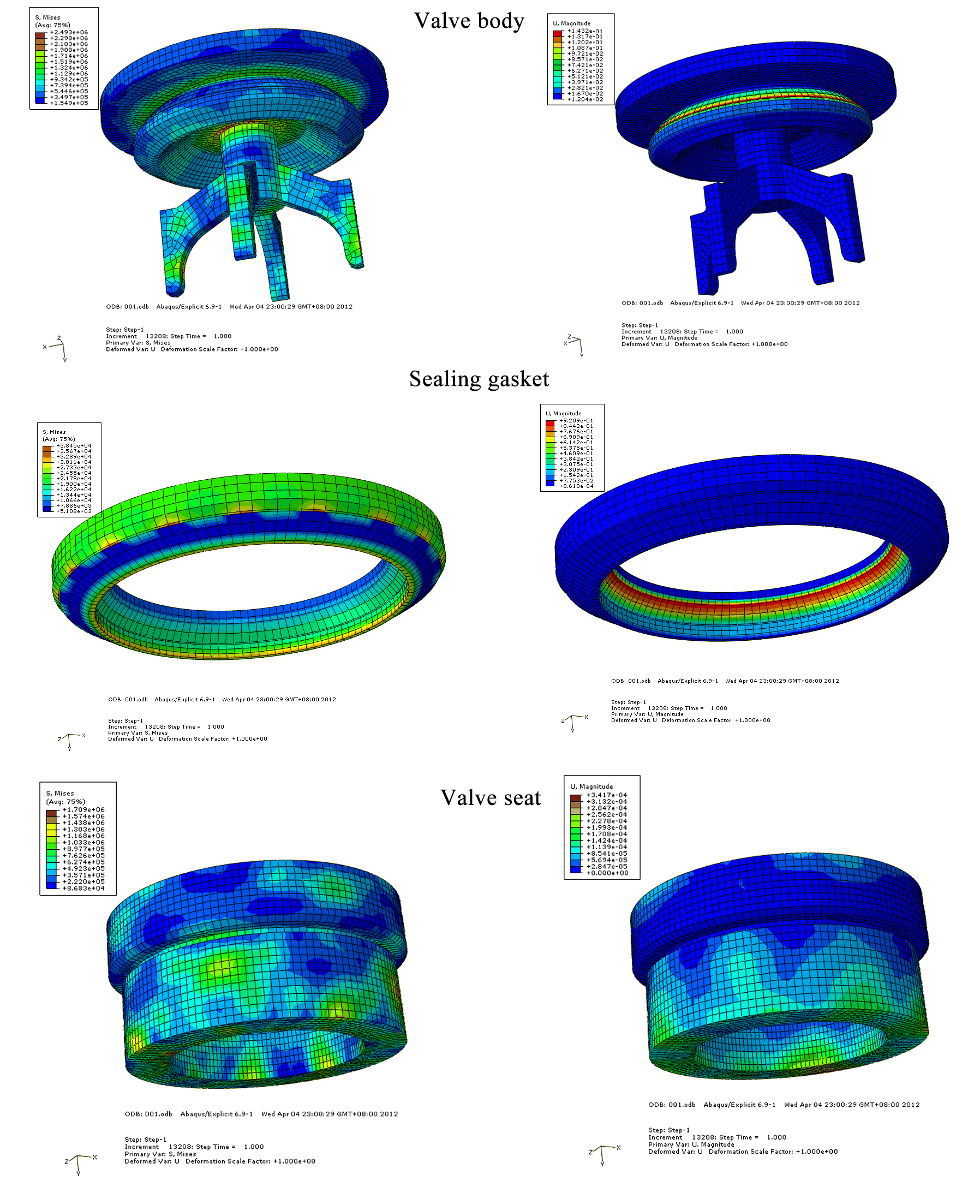

Supplement: S6 Fig — (TIF) [file pone.0140396.s006.tif]
