# Supplementary material for: Dynamic Behavior of Reciprocating Plunger Pump Discharge Valve Based on Fluid Structure Interaction and Experimental Analysis
Source: PLoS One. 2015 Oct 21;10(10):e0140396. doi: 10.1371/journal.pone.0140396 (PMC4619497; doi:10.1371/journal.pone.0140396)
Supplement: S1 Table — (DOCX) [file pone.0140396.s008.docx]

Tab. The calibration of displacement sensors standard zero

| Displacement  (mm) | Voltage  (V) | Displacement  (mm) | Voltage  (V) | Displacement  (mm) | Voltage  (V) |
| --- | --- | --- | --- | --- | --- |
| 0 | 0 | 51 | 1.76 | 102 | 3.43 |
| 3 | 0.11 | 54 | 1.85 | 105 | 3.52 |
| 6 | 0.22 | 57 | 1.96 | 108 | 3.64 |
| 9 | 0.33 | 60 | 2.06 | 111 | 3.73 |
| 12 | 0.42 | 63 | 2.15 | 114 | 3.83 |
| 15 | 0.52 | 66 | 2.25 | 117 | 3.94 |
| 18 | 0.64 | 69 | 2.35 | 120 | 4.05 |
| 21 | 0.74 | 72 | 2.45 | 123 | 4.13 |
| 24 | 0.84 | 75 | 2.55 | 126 | 4.24 |
| 27 | 0.95 | 78 | 2.65 | 129 | 4.35 |
| 30 | 1.06 | 81 | 2.75 | 132 | 4.43 |
| 33 | 1.15 | 84 | 2.84 | 135 | 4.53 |
| 36 | 1.26 | 87 | 2.96 | 138 | 4.63 |
| 39 | 1.36 | 90 | 3.05 | 141 | 4.72 |
| 42 | 1.46 | 93 | 3.14 | 144 | 4.82 |
| 45 | 1.55 | 96 | 3.24 | 147 | 4.92 |
| 48 | 1.66 | 99 | 3.34 | 150 | 5.03 |
