# Supplementary material for: Dynamic Behavior of Reciprocating Plunger Pump Discharge Valve Based on Fluid Structure Interaction and Experimental Analysis
Source: PLoS One. 2015 Oct 21;10(10):e0140396. doi: 10.1371/journal.pone.0140396 (PMC4619497; doi:10.1371/journal.pone.0140396)
Supplement: S2 Table — (DOCX) [file pone.0140396.s009.docx]

Tab. The stress and strain values of pump valve components under different seating velocity for the optimized spring stiffness and valve body quality

|  | Seating velocity  (0.4m/s) | | Seating velocity  (0.5m/s) | | Seating velocity  (0.6m/s) | | Seating velocity  (1m/s) | | allowable stress  (MPa) |
| --- | --- | --- | --- | --- | --- | --- | --- | --- | --- |
|  | Stress  (MPa) | Strain  (mm) | Stress  (MPa) | Strain  (mm) | Stress  (MPa) | Strain  (mm) | Stress  (MPa) | Strain  (mm) |  |
| Valve body | 0.88 | 0.138 | 3.7 | 0.139 | 4.4 | 0.139 | 5.2 | 0.143 | ≥850 |
| Sealing  gasket | 0.024 | 0.88 | 0.029 | 0.88 | 0.032 | 0.89 | 0.038 | 0.92 | 0.284971 |
| Valve seat | 0.5 | 7.3e-5 | 0.59 | 8.96e-5 | 1.2 | 1.2e-4 | 1.7 | 3.4e-4 | ≥850 |
